# Electrical stimulation of renal nerves for modulating urine glucose excretion in rats

**DOI:** 10.1186/s42234-018-0008-5

**Published:** 2018-05-29

**Authors:** Ahmad A. Jiman, Kavaljit H. Chhabra, Alfor G. Lewis, Paul S. Cederna, Randy J. Seeley, Malcolm J. Low, Tim M. Bruns

**Affiliations:** 1 Department of Biomedical Engineering, University of Michigan, Ann Arbor, MI USA; 2 Biointerfaces Institute, University of Michigan, Ann Arbor, MI USA; 3 Department of Molecular and Integrative Physiology, University of Michigan, Ann Arbor, MI USA; 4 Department of Surgery, University of Michigan, Ann Arbor, MI USA; 5 Department of Surgery, Plastic Surgery Section, Michigan Medicine, Ann Arbor, MI USA

**Keywords:** Electrical stimulation, Kidney, Renal nerve, Glucose, Urine, Glycosuria

## Abstract

**Background:**

The role of the kidney in glucose homeostasis has gained global interest. Kidneys are innervated by renal nerves, and renal denervation animal models have shown improved glucose regulation. We hypothesized that stimulation of renal nerves at kilohertz frequencies, which can block propagation of action potentials, would increase urine glucose excretion. Conversely, we hypothesized that low frequency stimulation, which has been shown to increase renal nerve activity, would decrease urine glucose excretion.

**Methods:**

We performed non-survival experiments on male rats under thiobutabarbital anesthesia. A cuff electrode was placed around the left renal artery, encircling the renal nerves. Ureters were cannulated bilaterally to obtain urine samples from each kidney independently for comparison. Renal nerves were stimulated at kilohertz frequencies (1–50 kHz) or low frequencies (2–5 Hz), with intravenous administration of a glucose bolus shortly into the 25–40-min stimulation period. Urine samples were collected at 5–10-min intervals, and colorimetric assays were used to quantify glucose excretion and concentration between stimulated and non-stimulated kidneys. A Kruskal-Wallis test was performed across all stimulation frequencies (α = 0.05), followed by a post-hoc Wilcoxon rank sum test with Bonferroni correction (α = 0.005).

**Results:**

For kilohertz frequency trials, the stimulated kidney yielded a higher average total urine glucose excretion at 33 kHz (+ 24.5%; *n* = 9) than 1 kHz (− 5.9%; *n* = 6) and 50 kHz (+ 2.3%; *n* = 14). In low frequency stimulation trials, 5 Hz stimulation led to a lower average total urine glucose excretion (− 40.4%; *n* = 6) than 2 Hz (− 27.2%; *n* = 5). The average total urine glucose excretion between 33 kHz and 5 Hz was statistically significant (*p* < 0.005). Similar outcomes were observed for urine flow rate, which may suggest an associated response. No trends or statistical significance were observed for urine glucose concentrations.

**Conclusion:**

To our knowledge, this is the first study to investigate electrical stimulation of renal nerves to modulate urine glucose excretion. Our experimental results show that stimulation of renal nerves may modulate urine glucose excretion, however, this response may be associated with urine flow rate. Future work is needed to examine the underlying mechanisms and identify approaches for enhancing regulation of glucose excretion.

## Background

Diabetes mellitus is a chronic progressive disease that requires continuous monitoring and medical care to prevent the development of severe complications (American Diabetes Association (ADA) 2018). Medications for diabetic management are numerous and have different mechanisms of action (Chatterjee and Davies 2015). Recently, sodium-glucose co-transporter 2 (SGLT-2) inhibitors were approved by the US Food and Drug Administration (FDA) for patients with type 2 diabetes. SGLT-2 inhibitors prevent the activity of SGLT-2 transporters in the renal proximal tubule, thereby reducing glucose reuptake by the kidneys and increasing glucose excretion into urine (Lew and Wick 2015). Despite the progress in the development of diabetic medications, many lose their effectiveness over time, which makes achieving blood glucose control targets difficult for many diabetic patients (Blak et al. 2012; Khunti et al. 2013; Ali et al. 2013). Furthermore, sustained patient adherence to these diabetic medications in a lifelong therapy is a major challenge (García-Pérez et al. 2013; Sabaté 2003). Therefore, there is a crucial need for alternative diabetic therapies that overcome these pharmaceutical limitations.

In recent years, a global interest has emerged for catheter-based renal denervation as a potential treatment for drug-resistant hypertension (Pan et al. 2015; Bhatt et al. 2014). Early clinical trials of renal denervation showed significant blood pressure improvements (Esler et al. 2010; Krum et al. 2009). Interestingly, renal denervation was also associated with significant decreases in blood glucose levels (Mahfoud et al. 2011; Witkowski et al. 2011). Renal denervation studies in animals align with the observed blood glucose control improvements reported in clinical trials (Rafiq et al. 2015; Iyer et al. 2016). Furthermore, a recent study reported that mutant (neuronal POMC-deficient) mice showed improved capability for tolerating high blood glucose levels by exaggerating urine glucose excretion (glycosuria) compared to wild-type mice at similar induced blood glucose concentrations (Chhabra et al. 2016). A following study determined that the observed glycosuria and improved glucose tolerance were a result of reduced activity in renal sympathetic nerves (Chhabra et al. 2017). A non-pharmaceutical and reversible approach that has emerged in recent years for reducing nerve activity is kilohertz frequency stimulation, which has demonstrated nerve conduction block on multiple types of nerves (Kilgore and Bhadra 2014; Joseph and Butera 2009; Joseph and Butera 2011). We hypothesized that kilohertz frequency stimulation (1–50 kHz) on renal nerves would attain similar results to renal denervation and induce urine glucose excretion.

Several studies have successfully influenced renal nerve activity in humans and animals by applying electrical stimulation. Electrical stimulation of renal nerves with an intra-arterial catheter electrode demonstrated increased blood pressure, and was considered as a method for locating suitable renal denervation targets for the treatment of drug-resistant hypertensive patients (Chinushi et al. 2013; Madhavan et al. 2014; Gal et al. 2015). Direct stimulation of renal nerves in rats using wire hook electrodes at low frequencies (0.5–10 Hz) showed increased renin secretion and water reabsorption, and decreased renal blood flow and sodium excretion responses (DiBona and Kopp 1997; DiBona and Sawin 1982; Bello-Reuss et al. 1976; Hermansson et al. 1981; Van Vliet et al. 1991). Sodium and glucose reabsorption are partially associated due to the presence of sodium-glucose co-transporters (SGLTs) in the renal proximal tubule (Mather and Pollock 2011). Our hypothesis was that direct stimulation of renal nerves at low frequencies (0.5–10 Hz) would decrease urine glucose excretion.

Therapies that directly alter neural activity (neuromodulation) are commonly prescribed as treatments for a variety of conditions (Krames et al. 2009; Famm et al. 2013). Gastric electrical stimulation is used to help patients with delayed stomach-emptying of solid foods (gastroparesis), which is commonly observed in patients with diabetes (Abell et al. 2003). Vagal nerve block (vBloc) therapy was recently approved by the FDA for certain patients with morbid obesity (Apovian et al. 2017). Clinical trials on vBloc therapy reported improvements in blood glucose control for patients with obesity and type 2 diabetes but were not sustained after 24 months (Herrera et al. 2017). Despite the success of neuromodulation therapies, to our knowledge, no clinical studies have investigated organ-targeted neuromodulation as a treatment approach for diabetes. In this study, we investigated modulation of urine glucose excretion with kilohertz and low frequency stimulation on renal nerves.

## Methods

All experimental procedures were approved by the University of Michigan Institutional Animal Care and Use Committee (IACUC).

### Animals and housing

Rats have a similar urinary system to humans and rat renal nerves have been visualized by several research groups (Stocker and Muntzel 2013; Miki et al. 2002). Non-survival, anesthetized experiments were performed on 24 male 290–550 g Long-Evans and Sprague-Dawley rats (Charles Rivers Laboratories, Wilmington, MA, USA). All animals were housed in ventilated cages under controlled temperature, humidity, and photoperiod (12-h light/dark cycle). The animals were provided with laboratory chow (5L0D, LabDiet, St. Louis, MO, USA) and tap water ad libitum.

### Experimental preparation

For anesthesia, a single dose of thiobutabarbital sodium salt hydrate (Inactin, T133-1G, Sigma-Aldrich Corp., St. Louis, MO, USA) was injected intraperitoneally (110 mg/kg BW). Thiobutabarbital is commonly used in renal studies and is known to preserve renal function during anesthesia (Walter et al. 1989; Sohtell et al. 1983). Rats were placed on a heating pad (ReptiTherm, Zoo Med Laboratories Inc., San Luis Obispo, CA, USA) and temperature was monitored through a rectal temperature sensor (SurgiVet, Smiths Medical, Norwell, MA, USA). Under a dissection microscope (Lynx EVO, Vision Engineering Inc., New Milford, CT, USA), a midline cervical incision was made and the jugular vein was cannulated with polyethylene tubing (BTPE-50, Instech Laboratories Inc., Plymouth Meeting, PA, USA). Through the jugular vein, 0.9% NaCl (saline), equivalent to 10% body weight, was infused over 30 min, and then followed by a continuous infusion of 0.2 mL/min using a syringe pump (NE-1000, New Era Pump Systems Inc., Farmingdale, NY, USA) (Bello-Reuss et al. 1976). A tracheotomy was performed to ensure a clear airway. Ureters were cannulated bilaterally with polyethylene tubing (BTPE-10, Instech Laboratories Inc., Plymouth Meeting, PA, USA) to obtain urine samples from each kidney independently. The left kidney was exposed through a midline abdominal incision. Fat and connective tissue surrounding the kidney were separated using cotton-tipped applicators to further expose the kidney and renal artery. A bipolar nerve cuff electrode (1.00 mm inner-diameter, 100 μm platinum contacts, Microprobes for Life Science, Gaithersburg, MD, USA) was placed around the renal artery, encircling renal nerves that run along the artery (Stocker and Muntzel 2013; Miki et al. 2002). Care was taken to not damage the renal nerve branches and to not occlude blood flow in the renal artery. To ensure that the renal nerves were intact, biphasic stimulation pulses at 10 Hz, 10 V were applied for approximately 1 min through the nerve cuff electrode. This resulted in temporary kidney ischemia, which was confirmed by the observation of kidney surface blanching (Hermansson et al. 1981; Yao et al. 2014). This stimulation-driven ischemia occurred in all the experiments in which we performed the test (*n* = 18). Prior to implant, electrode impedance measurements (4.77 ± 1.53 kΩ) were taken using an impedance tester (nanoZ, White Matter LLC, Seattle, WA, USA) at 1 kHz in saline to confirm functionality of the nerve cuff electrode.

### Electrical stimulation

The nerve cuff electrode placed on the renal nerves was connected to an isolated pulse stimulator (Model 4100, A-M Systems, Loop Sequim, WA, USA). For kilohertz frequency stimulation, a function generator (33220A, Agilent Technologies, Santa Clara, CA, USA) was connected to the isolated pulse stimulator to generate sinusoidal waveforms at 1, 33 or 50 kHz. The stimulation amplitude was fixed at 15 V, which has been shown to provide nerve conduction block for all selected frequencies on unmyelinated nerves (Joseph and Butera 2009; Joseph and Butera 2011). For low frequency stimulation, the isolated pulse stimulator generated biphasic pulses at 2 or 5 Hz. The stimulation amplitude and pulse width was fixed at 10 V and 0.5 msec, respectively, which is above the activation threshold for rat C-fibers using cuff electrodes (Woodbury and Woodbury 1990). The stimulation frequencies were randomly ordered between trials across all experiments to mitigate sequential effects.

### Experimental protocol

**Fig. 1:**
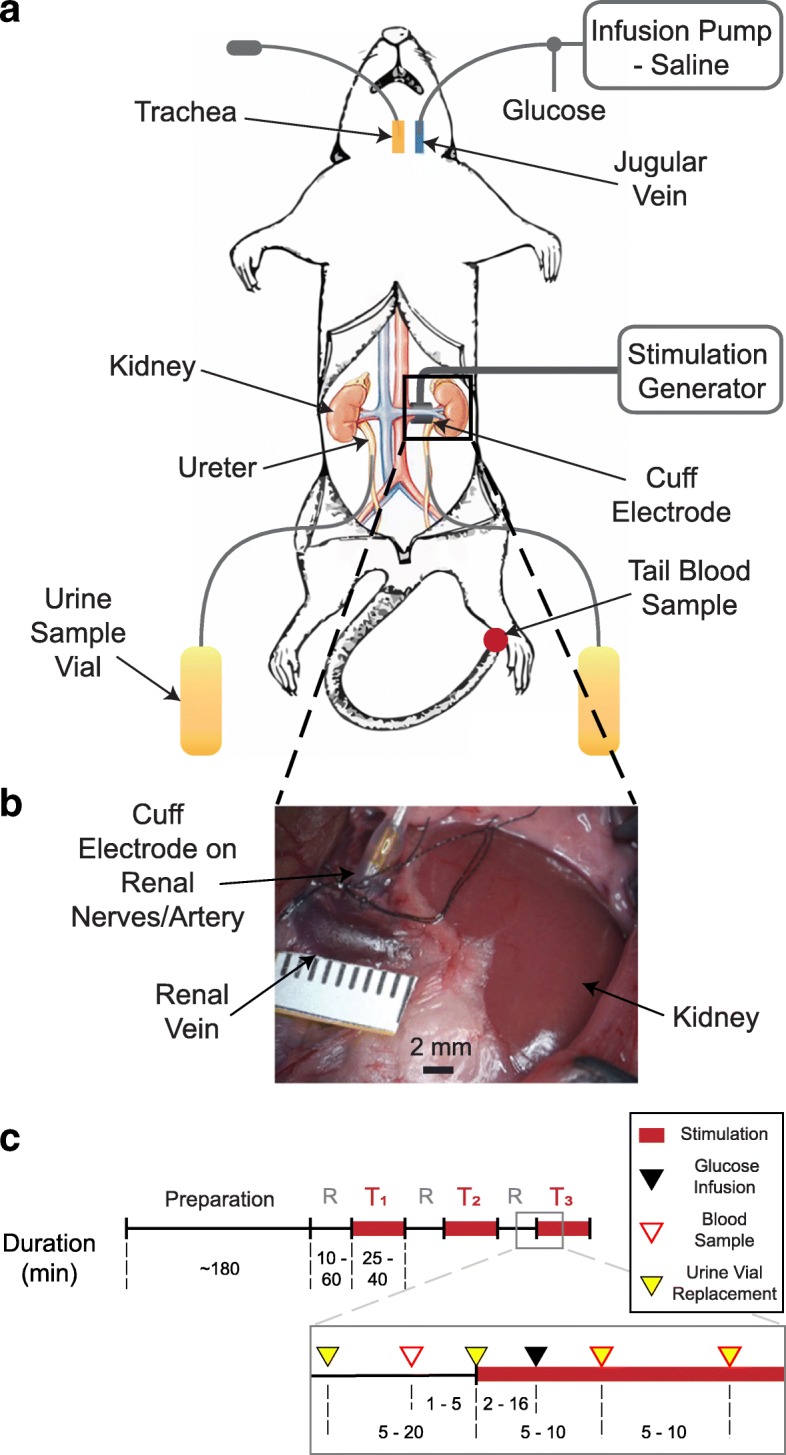
Experimental setup diagram and protocol timeline. **a** Experimental setup: Jugular vein was cannulated for saline and glucose infusion. Nerve cuff electrode was placed on renal nerves of the left kidney and connected to a stimulation generator. Ureters were cannulated bilaterally, and urine samples were collected in sampling vials. **b** Nerve cuff electrode was placed around the renal artery, encapsulating the renal nerve branches that run along the renal artery. **c** Timeline for experimental protocol: Each experiment consisted of 1–3 stimulation trials (T_1_-T_3_), with a rest period (R) before each trial. A glucose bolus was infused in each trial. Blood glucose measurements and urine samples were obtained periodically throughout the trials

After completion of surgery, a stabilization period of 10–60 min was provided. In each experiment, 1–3 trials with different stimulation frequencies were applied on the nerve cuff electrode. Stimulation was applied at the start of a trial and remained on for 25–40 min. To elevate blood glucose levels beyond the expected renal threshold for glucose excretion (400 mg/dL) (Liang et al. 2012), a 0.30–1.00 g bolus dose of glucose (50% Dextrose Injection USP, Hospira Inc., Lake Forest, IL, USA) was delivered through the jugular vein at 2–16 min into each trial. To confirm blood glucose increase and to monitor blood glucose levels over time, drops of blood (< 0.1 mL) from a tail cut were used to obtain blood glucose concentration measurements using a glucometer (AlphaTRAK 2, Abbott, Abbott Park, IL, USA) before glucose infusion and every 5–10 min after glucose infusion. Urine samples from each kidney were collected in pre-weighed sampling tubes (3448, Thermo Fisher Scientific, Waltham, WA, USA) at 5–10-min intervals. Ten minutes after the end of a trial, blood glucose measurements were expected to be around baseline levels. If not, a longer washout period was provided to the rat before proceeding to the next experimental trial. The collected urine samples were weighed on a scale (AE 160, Mettler Toledo, Columbus, OH, USA) for volume estimations (1 μL/mg). Urine glucose concentrations were measured using colorimetric assays (10009582, Cayman Chemical, Ann Arbor, MI, USA). The experimental setup and protocol timeline are summarized in Fig. 1.

From the urine sample volumes and glucose concentration measurements, the total urine glucose excretion (UGE) was calculated and compared between the stimulated and non-stimulated kidney [∆UGE = (UGE_stimulated_ – UGE_non-stimulated_)/UGE_non-stimulated_ × 100] for each trial. For urine glucose concentration (UGC) and urine flow rate (UFR), the area under the curve (AUC) was calculated for each trial by trapezoidal numerical integration and compared between the kidneys in a similar manner as UGE. From blood glucose concentration (BGC) values, a BGC decrease rate (BGCDR) was obtained by calculating the linear regression slope of BGC values starting approximately 10 min after the glucose bolus infusion and ending with the final value in the trial. The glucometer was unable to read blood glucose concentrations above 750 mg/dL, which occasionally occurred during the first 10 min after a glucose bolus infusion. Therefore, BGC values within 10 min after a glucose bolus infusion were excluded in BGCDR calculations for all stimulation trials.

### Statistical analysis

Across all experiments, data sets did not follow a normal distribution (confirmed by one-sample Kolmogorov-Smirnov test). Therefore, a non-parametric Kruskal-Wallis test was performed to measure statistical significance across stimulation frequencies. Statistical significance was considered at *p* < 0.05. A two-sided Wilcoxon rank sum test was then applied between pairs of stimulation frequencies. The significance level (α) was adjusted according to a Bonferroni correction, where α was divided by the number of stimulation pairs (10). Thus, statistical significance for the Wilcoxon rank sum test was considered at *p* < 0.005. All data analysis and statistical tests were performed using MATLAB software (R2014b, MathWorks, Natick, MA, USA).

## Results

Across the 24 experiments on male rats, we performed stimulation trials at kilohertz frequencies (1 kHz [*n* = 6], 33 kHz [*n* = 9] and 50 kHz [*n* = 14]) and low frequencies (2 Hz [*n* = 5] and 5 Hz [n = 6]). We obtained measurements of urine glucose excretion, urine glucose concentration, urine flow rate, and blood glucose concentration in each trial.

### Urine glucose excretion

**Fig. 2:**
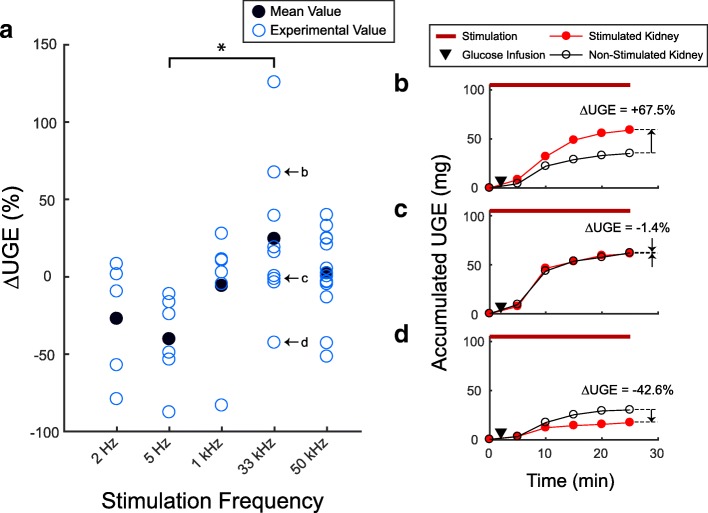
Changes in urine glucose excretion. **a** The percentage difference in urine glucose excretion between the stimulated and non-stimulated kidney (∆UGE) at the applied stimulation frequencies. Stimulation frequency had a statistically significant main effect (Kruskal-Wallis test, *p* < 0.05), with one within-frequency comparison being significant (5 Hz and 33 kHz, post-hoc Wilcoxon rank sum test, * = *p* < 0.005). **b** Representative stimulation trial at 33 kHz that showed an increase in UGE. **c** Representative stimulation trial at 33 kHz that showed no apparent effect on UGE. **d** Representative stimulation trial at 33 kHz that showed a decrease in UGE

Glucose excretion was analyzed and compared between the urine samples obtained from the stimulated and non-stimulated kidneys. The percentage difference of urine glucose excretion (∆UGE) between the stimulated and non-stimulated kidneys for all stimulation frequencies are shown in Fig. 2a. Overall, stimulation frequency had a statistically significant effect on ∆UGE (Kruskal-Wallis test, *p* < 0.05). In kilohertz frequency trials, 33 kHz yielded a higher average ∆UGE (+ 24.5%; *n* = 9) than 1 kHz (− 5.9%; *n* = 6) and 50 kHz (+ 2.3%; *n* = 14). In low frequency trials, 5 Hz stimulation led to a lower average ∆UGE (− 40.4%; n = 6) than 2 Hz (− 27.2%; *n* = 5). Statistical significance only occurred between the ∆UGE of 33 kHz and 5 Hz trials (Wilcoxon rank sum test, *p* < 0.005). Stimulation at kilohertz frequencies met our hypothesis of increased UGE in 14 trials (48.2%), had no apparent effect (|∆UGE| < 5%) in 10 trials (34.5%), and showed a decrease in UGE in 5 trials (17.2%) out of the 29 total kilohertz frequency trials. In low frequency stimulation trials, we observed a decrease of UGE in 9 trials (81.8%), no apparent effect in 1 trial (9.1%), and an increase of UGE in 1 trial (9.1%) out of 11 trials in total. Examples of stimulation trials at 33 kHz that displayed an increase, no apparent effect, or a decrease in UGE are shown in Fig. 2b-d.

### Urine glucose concentration

**Fig. 3:**
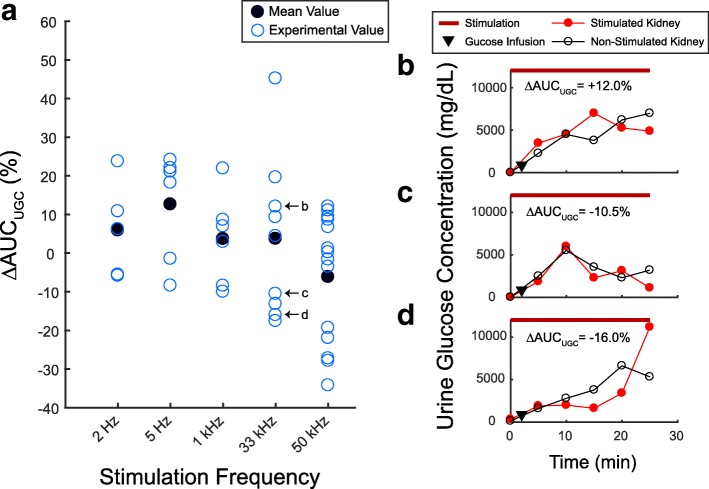
Changes in urine glucose concentration. **a** The percentage difference between the area under the curve for urine glucose concentration of the stimulated and non-stimulated kidney (∆AUC_UGC_) at the applied stimulation frequencies. **b** Urine glucose concentration (UGC) measurements for the trial shown in Fig. 2b. **c** UGC measurements for the trial shown in Fig. 2c. **d** UGC measurements for the trial shown in Fig. 2d

The urine glucose concentration (UGC) differences between the urine samples obtained from the stimulated and non-stimulated kidneys at all stimulation frequencies are shown in Fig. 3a. The average UGC difference was + 5.9% at 2 Hz (*n* = 5), + 12.6% at 5 Hz (*n* = 6), + 3.7% at 1 kHz (n = 6), + 3.7% at 33 kHz (*n* = 9), and − 6.2% at 50 kHz (*n* = 14). Stimulation frequency did not have an overall significant effect on UGC (Kruskal-Wallis test, *p* = 0. 2365).

### Urine flow rate

**Fig. 4:**
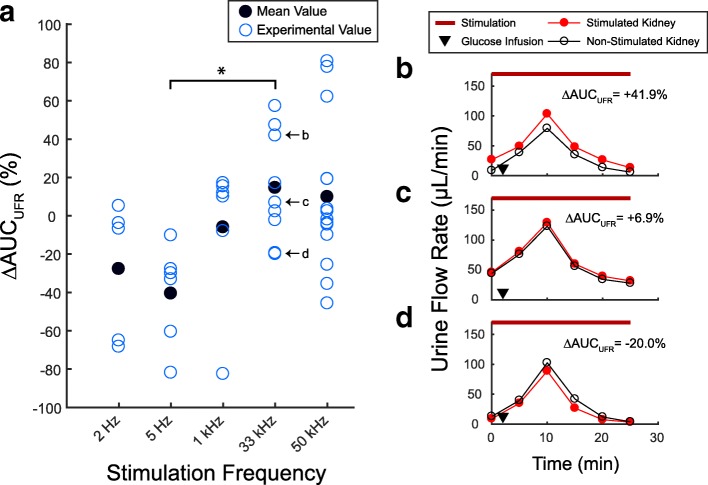
Changes in urine flow rate. **a** The percentage difference between the area under the curve for urine flow rate of the stimulated and non-stimulated kidney (∆AUC_UFR_) at the applied stimulation frequencies. Stimulation frequency had a significant main effect (Kruskal-Wallis test, *p* < 0.05), with 5 Hz and 33 kHz trials significantly different from each other (post-hoc Wilcoxon rank sum test, * = *p* < 0.005). **b** Urine flow rate (UFR) measurements for the trial shown in Fig. 2b. **c** UFR measurements for the trial shown in Fig. 2c. **d** UFR measurements for the trial shown in Fig. 2d

The urine flow rate (UFR) differences between the urine samples obtained from the stimulated and non-stimulated kidneys at all stimulation frequencies are shown in Fig. 4a. The average UFR difference was − 27.7% at 2 Hz (*n* = 5), − 40.6% at 5 Hz (*n* = 6), − 6.0% at 1 kHz (*n* = 6), + 14.6% at 33 kHz (*n* = 9), and + 9.8% at 50 kHz (*n* = 14). Stimulation frequency had a statistically significant main effect on UFR (Kruskal-Wallis test, *p* < 0.05), with trials at 33 kHz and 5 Hz significantly different from one another (post-hoc Wilcoxon rank sum test, *p* < 0.005).

### Blood glucose concentration

**Fig. 5:**
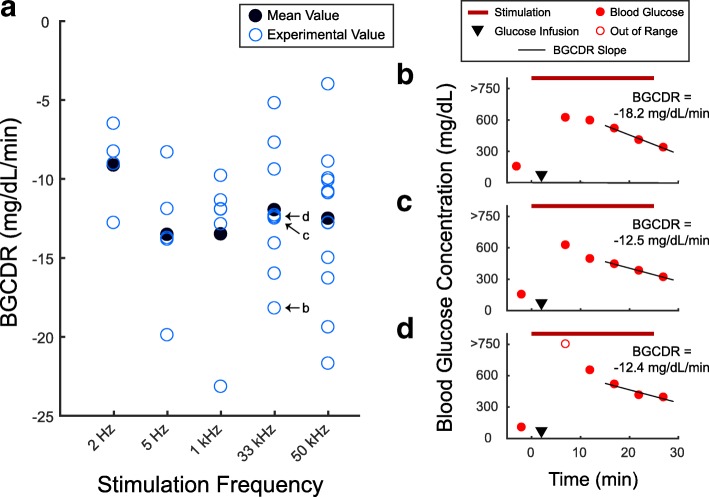
Changes in blood glucose concentration. **a** The blood glucose concentration decrease rate (BGCDR) at the applied stimulation frequencies. **b** Blood glucose concentration (BGC) measurements and BGCDR (slope) for the trial shown in Fig. 2b. **c** BGC and BGCDR measurements for the trial shown in Fig. 2c. **d** BGC and BGCDR measurements for the trial shown in Fig. 2d. BGC measurements above 750 mg/dL were not available due to the limitations of the glucometer

The blood glucose concentration decrease rates (BGCDRs) during stimulation at all frequencies are shown in Fig. 5a. The average BGCDR was − 9.1 mg/dL/min at 2 Hz (*n* = 4), − 13.5 mg/dL/min at 5 Hz (*n* = 5), − 13.5 mg/dL/min at 1 kHz (*n* = 6), − 12.0 mg/dL/min at 33 kHz (*n* = 9), and − 12.5 mg/dL/min at 50 kHz (*n* = 13). No statistically significant main effect occurred across all stimulation frequencies (Kruskal-Wallis test, *p* = 0.4708). BGCDR at some stimulation trials [2 Hz (*n* = 1), 5 Hz (*n* = 1) and 50 kHz (*n* = 1)] were not calculated due to insufficient BGC values.

## Discussion

The aim of this study was to investigate modulation of urine glucose excretion by electrical stimulation of renal nerves. We hypothesized that stimulation of renal nerves at kilohertz frequencies (1–50 kHz) would increase urine glucose excretion (UGE), while low frequency stimulation (2–5 Hz) would decrease UGE. Although stimulation at kilohertz frequencies did not always lead to an increase in UGE, 33 kHz showed a notable average increase in UGE in accordance with our hypothesis. In contrast, low frequency stimulation typically showed a decrease in UGE, with the strongest effect observed at 5 Hz stimulation (Fig. 2). To our knowledge, this study is the first to demonstrate influence of electrical stimulation of renal nerves on glucose excretion.

The average differences in UGE were similar to the average differences observed in urine flow rate (UFR), as shown in Fig. 4. This associated response may suggest that either UGE or UFR was the primary effect of stimulation, while the other was a secondary response. Previous studies that applied stimulation of renal nerves at low frequencies observed a 25–52% reduction in UFR (Bello-Reuss et al. 1976; Pontes et al. 2015). Those percentages align with the average reduction of UFR we observed at low frequency stimulation (28% at 2 Hz, 41% at 5 Hz), suggesting that UFR may be the primary response of stimulation at low frequencies. On the other hand, we observed an increase in UFR at 33 and 50 kHz stimulation. To our knowledge, no studies have reported an increase in UFR by stimulation of renal nerves. Although it is possible that changes in UFR may have directly led to corresponding changes in UGE, the primary response of UFR or UGE to stimulation at kilohertz frequencies cannot be determined in this study. UFR and UGE are normally associated, as increased urination is a common adverse event in diabetic patients treated with sodium-glucose co-transporter 2 (SGLT2) inhibitors that primarily increase urine glucose excretion (Seufert 2015; Wilding 2014). Additional studies are required to distinguish the glucose excretion and urine flow effects for stimulation of renal nerves.

Stimulation of renal nerves did not have a clear effect on urine glucose concentration (UGC), as no statistical significance occurred across stimulation frequencies (Fig. 3). Furthermore, we did not observe a clear difference between kilohertz or low frequency stimulation on the decrease rate for blood glucose concentration (BGC) after infusion of a glucose bolus (Fig. 5). Typically, BGC would reach a peak value within the first 10 min after glucose bolus infusion. Then, BGC values would gradually decrease and return to around baseline values at 30–40 min after the glucose infusion, regardless of the stimulation parameters. The variation in the sample size of the stimulation frequency groups may have also contributed to these unclear responses. Modifications and improvements in experimental design may be necessary to capture clear and consistent responses to stimulation of renal nerves.

Renal nerve branches are distributed around the renal artery in a plexus form. Ultrastructural studies using electron microscopy techniques have shown that renal nerve fibers innervate epithelial cells of proximal tubules, the glucose reabsorption region of the kidney (Mather and Pollock 2011; Muller and Barajas 1972; Luff et al. 1992). Although studies have examined the distribution of renal nerves around the renal artery (Maeda et al. 2014; Sakakura et al. 2014; van Amsterdam et al. 2016), we could not determine the renal nerve branches that innervate the proximal tubules in this study. Therefore, we utilized a cuff electrode with the purpose of encircling all the renal nerve branches surrounding the renal artery. In order to place a cuff electrode, the renal artery was isolated by removing adjacent connective tissue that may have contained fine renal nerve branches. Although we ensured that the renal nerves were moderately intact by observing temporary kidney surface blanching at 10 Hz stimulation (Hermansson et al. 1981; Yao et al. 2014), the variations in connective tissue removal and relative shifts in the electrode placement along the renal artery across experiments may have contributed to the variability of our outcome results. This inconsistency in outcomes has also been observed in renal denervation studies, where conflicting results were reported in clinical studies (Bhatt et al. 2014; Mahfoud et al. 2011; Witkowski et al. 2011). The reported variability is suspected to be from variations in ablation locations across renal denervation procedures performed in multiple centers (Mahfoud et al. 2014). Experimental improvements in electrode placement and the plexus-electrode interface may be required to obtain more consistent results.

An anatomical analysis in rats showed that 96% of renal nerve axons are unmyelinated C-fibers (DiBona et al. 1996). Although nerve conduction block experiments using kilohertz frequency stimulation have been typically performed using cuff electrodes encircling myelinated motor neurons while monitoring muscle tension for block validation (Kilgore and Bhadra 2014; Bhadra and Kilgore 2005), nerve block has also been demonstrated on purely unmyelinated fibers using suction electrodes and confirmed by direct recordings of action potential propagation (Joseph and Butera 2009). In this study, the amplitude of sinusoidal kilohertz frequency stimulation was fixed at 15 V, which is expected to be above the threshold for nerve conduction block at the selected frequencies (Joseph and Butera 2011; Bhadra and Kilgore 2005; Patel and Butera 2015). On the other hand, previous studies increased renal nerve activity by low frequency stimulation (Bello-Reuss et al. 1976; DiBona 2000). The stimulation amplitude and pulse width in this study at low frequencies was consistent at 10 V and 0.5 msec, respectively, which is above the activation threshold for rat C-fibers using cuff electrodes (Woodbury and Woodbury 1990). However, to validate the true presence of nerve conduction block or increased neural activity, multiple recording and stimulating electrodes must be placed along the renal nerves. Unfortunately, this was difficult to accomplish in this study due to our limited ability to expose and isolate the renal nerves (~ 2–4 mm), in addition to the anticipated noise contamination issues between adjacent stimulating and recording electrodes (Kilgore and Bhadra 2014). Additional experiments are required to examine the mechanism of action for stimulation of renal nerves.

The work presented here was a feasibility study to investigate glucose excretion modulation by stimulation of renal nerves. There are numerous limitations to this study. Although changes in UGE were observed in response to stimulation of renal nerves, this study does not provide any evidence on the underlying mechanisms for these changes. It is unknown if the observed changes in UGE were a consequence of changes in UFR, or directly related to the gluconeogenesis process or the glucose transport pathways in the proximal tubules that are innervated by renal nerves (Mather and Pollock 2011; Muller and Barajas 1972; Luff et al. 1992). Measurements of renal function, such as glomerular filtration rate, renal plasma flow and sodium excretion (Toto 1995; Phillips and Hamilton 1948) were not obtained in this feasibility study. The assessment of renal function is an absolute necessity for the progression of this research. The large variation in the results of this study may have been due to multiple reasons. In addition to the variability in electrode placement, the unilateral stimulation approach in this study may have provoked reno-renal reflexes, where the non-stimulated kidney modifies its activity based on changes in the stimulated kidney (Zanchetti et al. 1984). The possible presence of these reflexes may have altered the outcomes of this study. Further experiments with reno-renal reflex elimination procedures, such as bilateral stimulation or denervation of non-stimulated kidneys, may be necessary to obtain unhindered stimulation outcomes.

Although further experiments are required to examine the underlying mechanisms for stimulation of renal nerves, this study may introduce a new approach for regulation of glucose excretion. Recently approved medications for patients with type 2 diabetes are SGLT-2 inhibitors, which prevent the activity of glucose transporters in the kidney and lead to increased glucose excretion into urine (Lew and Wick 2015). Stimulation of renal nerves may provide an alternative treatment approach for glycemic control that avoids patient compliance issues typically seen with medications (Polonsky and Henry 2016).

## Conclusion

To our knowledge, this is the first study to investigate electrical stimulation of renal nerves to modulate urine glucose excretion. Our experimental results show that stimulation of renal nerves may modulate urine glucose excretion, however, this outcome may be associated with urine flow rate. Future work is needed to examine the underlying mechanisms and identify approaches for enhancing regulation of glucose excretion.

## Abbreviations

AUC: Area under the curve
BGC: Blood glucose concentration
BGCDR: Blood glucose concentration decrease rate
UFR: Urine flow rate
UGC: Urine glucose concentration
UGE: Urine glucose excretion
